# Muscular and Molecular Pathology Associated with SPATA5 Deficiency in a Child with EHLMRS

**DOI:** 10.3390/ijms22157835

**Published:** 2021-07-22

**Authors:** Frederik Braun, Andreas Hentschel, Albert Sickmann, Theodore Marteau, Swantje Hertel, Fabian Förster, Holger Prokisch, Matias Wagner, Saskia Wortmann, Adela Della Marina, Heike Kölbel, Andreas Roos, Ulrike Schara-Schmidt

**Affiliations:** 1 Centre for Neuromuscular Disorders, Centre for Translational Neuro- and Behavioral Sciences, Department of Pediatric Neurology, University Duisburg-Essen, 45147 Essen, Germany; Theodore.Marteau@uk-essen.de (T.M.); Swantje.Hertel@uk-essen.de (S.H.); Fabian.Foerster@uk-essen.de (F.F.); Adela.Dellamarina@uk-essen.de (A.D.M.); heike.koelbel@uk-essen.de (H.K.); Andreas.Roos@uk-essen.de (A.R.); Ulrike.Schara-Schmidt@uk-essen.de (U.S.-S.); 2 Leibniz-Institut für Analytische Wissenschaften—ISAS e.V., 44139 Dortmund, Germany; andreas.hentschel@isas.de (A.H.); albert.sickmann@isas.de (A.S.); 3 Institute of Human Genetics, Klinikum Rechts der Isar, School of Medicine, Technical University of Munich, 81675 Munich, Germany; prokisch@helmholtz-muenchen.de (H.P.); Matias.Wagner@mri.tum.de (M.W.); s.wortmann@salk.at (S.W.); 4 Helmholtz Zentrum München—German Research Center for Environmental Health, Institute of Neurogenomics, 85764 Neuherberg, Germany; 5 Children’s Hospital of Eastern Ontario Research Institute, Ottawa, ON K1H 5B2, Canada

**Keywords:** EHLMRS, SPATA5, mitochondrial disorder, muscle proteomics, myopathology

## Abstract

Mutations in the *SPATA5* gene are associated with epilepsy, hearing loss and mental retardation syndrome (EHLMRS). While *SPATA5* is ubiquitously expressed and is attributed a role within mitochondrial morphogenesis during spermatogenesis, there is only limited knowledge about the associated muscular and molecular pathology. This study reports on a comprehensive workup of muscular pathology, including proteomic profiling and microscopic studies, performed on an 8-year-old girl with typical clinical presentation of EHLMRS, where exome analysis revealed two clinically relevant, compound-heterozygous variants in *SPATA5*. Proteomic profiling of a quadriceps biopsy showed the dysregulation of 82 proteins, out of which 15 were localized in the mitochondrion, while 19 were associated with diseases presenting with phenotypical overlap to EHLMRS. Histological staining of our patient’s muscle biopsy hints towards mitochondrial pathology, while the identification of dysregulated proteins attested to the vulnerability of the cell beyond the mitochondria. Through our study we provide insights into the molecular etiology of EHLMRS and provide further evidence for a muscle pathology associated with SPATA5 deficiency, including a pathological histochemical pattern accompanied by dysregulated protein expression.

## 1. Introduction

**Figure 1:**
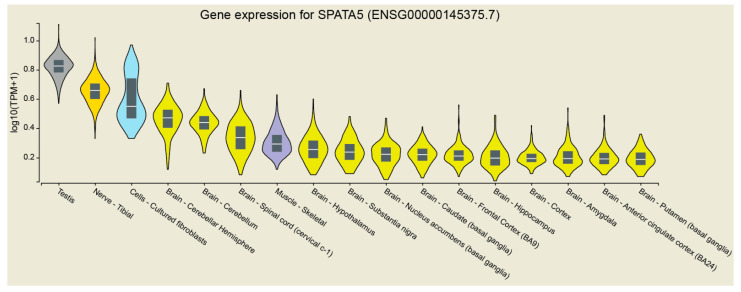
Gene expression for SPATA5.

The SPATA5 gene (NM_145207.2), located in the chromosomal region 4q28.1, encodes the spermatogenesis-associated protein (SPATA5, OMIM *613940) or spermatogenesis associated factor (SPAF), an 892 amino acid AAA Protein (ATPase associated with diverse activities). It is attributed a role in spermatogenesis and malignant conversion and shows mitochondrial localization. SPATA5 contains a putative mitochondrial matrix-targeting sequence and plays a role in mitochondrial morphogenesis during spermatogenesis [1]. There is ubiquitous tissue expression, including the testes, spleen, skin, intestines and brain [1,2]. Furthermore, in silico studies also show an expression in skeletal muscle (Figure 1).

The role of SPATA5 in mitochondrial integrity and function has been discussed in accordance with its involvement in synaptic development and plasticity, as well as developmental expression throughout the brain [2,3,4]. In functional studies, Puusepp et al. demonstrated its role in mitochondrial dynamics, causing an imbalance in mitochondrial fusion–fission rates, impaired energy production and short axons [5].

In contrast, there is incomplete knowledge on SPATA5-associated myopathology. Although morphological studies reveal that muscle biopsies have shown enlarged and abnormally shaped mitochondria, intermyofibrillar linear mitochondrial aggregates, a mild increase in subsarcolemmal and intermyofibrillar mitochondria, biochemical studies are limited and, thus far, have shown only reduced electron transport chain activity [2].

The epilepsy, hearing loss and mental retardation syndrome (EHLMRS, OMIM #616577) is caused by pathogenic biallelic variants in the SPATA5 gene and recent descriptions of cases (38 total) have led to the delineation of a coherent phenotype [6,7,8].

The EHLMRS was first described by Tanaka et al. and is associated with microcephaly, developmental delay, intellectual disability, hypotonia, spasticity, seizures, sensorineural hearing loss and cortical visual impairment. Most affected individuals are unable to sit or have no head control and have no, or minor, speech development, with their speech limited to just a few words. Brain affection is present, including cerebral atrophy, hypomyelination or a thin corpus callosum [2,6,8,9,10,11,12]. Dysmorphic facies with a depressed nasal ridge, broad eyebrows and retrognathia was described by Kurata et al., but does not seem to be shared with other cases in the literature [9]. 

We report on a comprehensive workup of muscular pathology, including proteomic profiling and microscopic studies, in an 8-year-old girl with typical clinical presentation of EHLMRS, where exome analysis revealed two pathogenic, compound-heterozygous variants in SPATA5.

## 2. Results

### 2.1. Clinical Findings

The patient is the first and only child of a non-consanguineous couple of European descent. Following an uncomplicated pregnancy, the spontaneous birth at term was uneventful [weight 3260 g (25 P., −0.67 z), length 49 cm (8 P., −1.39 z), head circumference 37 cm (93 P., 1.45 z), APGAR 9/10/10]. Postnatal brainstem audiometry showed sensorineural deafness, for which she received a hearing device before the age of two months. Hip luxation and Coxa valga antetorta, both present at birth, required multiple surgeries. Developmental milestones were delayed and, at one-and-a-half years of age, she could turn from the prone to supine position, but neither sit nor crawl and only produced single syllables. She continued to develop a global developmental delay, prominent axial hypotonia and spasticity, pronounced in the lower extremities [Gross Motor Function Classification System (GMFCS) Grade 4], as well as a dystonic movement disorder, pronounced in the hands. Deep tendon reflexes of the lower extremities were brisk. Cranial MRI at 6 years of age showed cortico-subcortical atrophy, predominantly posterior periventricular leukoencephalopathy, as well as a thin corpus callosum. Electroencephalograms were repeatedly abnormal and showed pathological findings, including sharp waves, while there is no clear clinical correlate.

Laboratory examinations for metabolic disorders were unremarkable, except for mildly elevated venous levels of lactic acid (preprandial 9.6 mg/dL, postprandial 27.6 mg/dL, normal range 5–20 mg/dL). This included normal results for ammonium, very long chain fatty acids (VLCFA), organic acids in urine, lysosomal enzymes, acylcarnitine profile, carnitine, mucopolysaccharides and oligosaccharides in urine, guanidine compounds in urine, amino acids, as well as a screening for congenital disorders of glycosylation (CDG). Creatine kinase (CK) levels in serum were normal.

**Figure 2:**
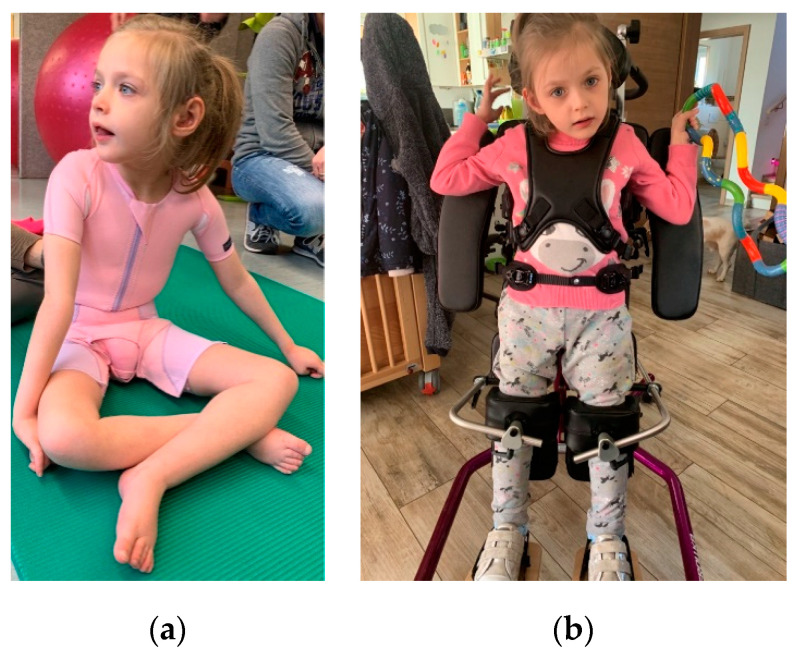
Patient’s photograph at 8 years of age: (**a**) patient sitting supported, displaying joint hypermobility, a depressed nasal ridge, retrognathia and large ears; (**b**) patient fixated in a standing aid, equivalent to best gross motor function, while the dystonic movement disorder is most pronounced in the hands.

At the last follow up, at 8 years of age, she was dystrophic and microcephalic [weight 19.5 kg (1 P., −2.40 z), length 105 cm (<1 P., −4.47 z), head circumference 49 cm (<1 P., −2.60 z)], could not sit unsupported, stand nor walk and only used one word. Additionally, there was mild joint hypermobility. To date, electrocardiogram and echocardiography results continue to be unremarkable. Our patient currently shows a mildly depressed nasal ridge, retrognathia and comparatively large ears (Figure 2).

### 2.2. Genetic Findings

Karyogram and array CGH analyses were unremarkable. The trio exome analysis revealed compound heterozygous variants in SPATA5 (NM_145207.2): c.[394C>T]; [989_991del], p.[Gln132Ter]; [Thr330del]. The paternal variant c.394C>T is predicted to lead to a premature stop of protein translation and is listed as pathogenic and the maternal variant c.989_991del, p.Thr330del, has been listed as likely to be pathogenic in ClinVar.

### 2.3. Muscle Biopsy Findings

**Figure 3:**
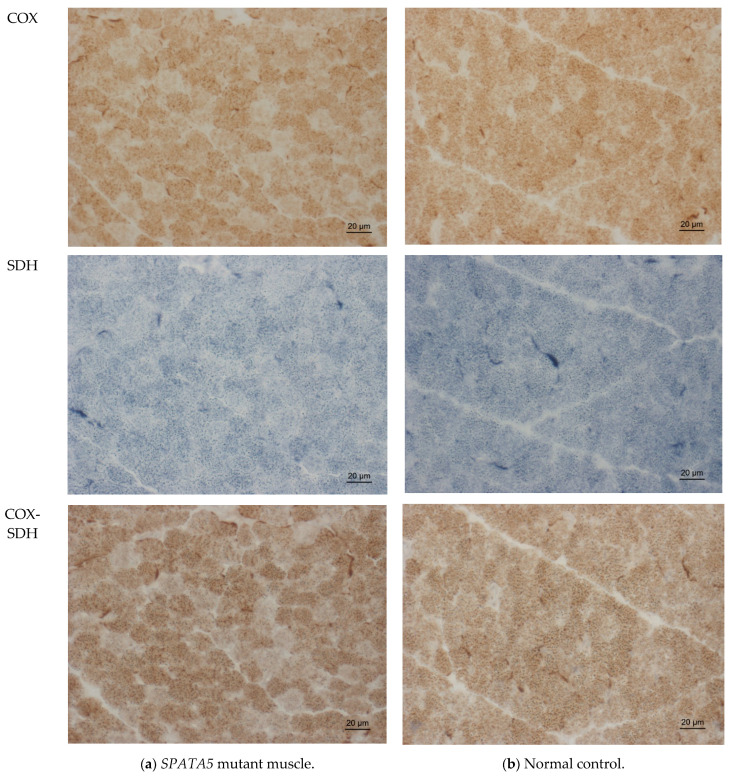
Enzyme histochemistry in SPATA5 mutant quadriceps muscle showing slightly reduced SDH reaction and no COX-negative fibers (**a**); comparison to normal control (**b**). COX, cytochrome c oxidase; SDH, succinate dehydrogenase.

Due to suspected mitochondriopathy, including muscular involvement, a muscle biopsy was performed at two years of age. Histological investigation of the muscle specimen taken from the right vastus lateralis muscle did not show any abnormalities, especially not in hematoxylin and eosin (HE) and Gomori trichrome stains. Results were normal for periodic acid-Schiff (PAS), nicotinamide adenine dinucleotide (NADH), as well as for ATPase 4.3 and ATPase 9.4 treatment (data not shown). A thorough immunohistological examination of the muscle specimen yielded normal results. An enzyme histochemistry disclosed reduced succinate dehydrogenase (SDH) reaction (Figure 3a) compared to a normal control (Figure 3b), but normal enzyme reaction for cytochrome c oxidase (COX) and combined COX-SDH (Figure 3a). 

An examination of respiratory chain enzymes hinted towards mitochondriopathy, with low activity within the reference range [Complex I 35.2 U/gNCP (ref. range 15.8–42.8) and 0.23 (relative to citrate synthase (CS), 0.17–0.56); Complex II/III 12.0 U/gNCP (6.0–25.0) and 0.08 (relative to CS, 0.08–0.45); Complex IV 185.5 U/gNCP (112–351) and 1.20 (relative to CS, 1.10–5.0); CS 155.0 U/gNCP (45–100); non-collagen protein (NCP) 5.1 g/L].

### 2.4. Proteomic Profiling

An examination of the proteomic profile performed on the muscle biopsy of the patient revealed the robust quantification of 1778 proteins (ProteomeXchange project accession #: PXD026182). 

Among these, we observed the significant up-regulation of 31 proteins and down-regulation of 51 proteins (Table S1), out of which 8, respectively 7, showed a mitochondrial localization (Table 1).

Filtering the proteomic signature for significantly dysregulated proteins with known involvement in specific diseases (using both UniProt and OMIM) resulted in a selection of associations with a phenotypical overlap to EHLMRS, or selected features in our patient. (Table 2) Out of the 31 significantly up-regulated proteins, 4 proteins have a known disease association. They are associated with primarily muscular disorders (PURA1, LAMA2), and other diseases with muscular involvement. This includes muscular weakness and lipid storage myopathy alongside a facial dysmorphism in glutaric academia IIB (ETFB) [13]. ECHA is known to be associated with multiple acyl-CoA dehydrogenase deficiency (MADD), myopathy, rhabdomyolysis and sensorimotor axonal neuropathy in mitochondrial trifunctional protein deficiency, and recurrent myalgia in long-chain 3-hydroxyacyl-CoA dehydrogenase (LCHAD) deficiency [13,14,15,16]. Overall, an association with fatty acid beta-oxidation, but also motor function and acetyl-CoA C-acyltransferase activity can be noted.

Out of the 51 significantly down-regulated proteins, 15 proteins have a known disease association. They are linked to a large variety of biological processes and molecular functions, and only some display a phenotypical overlap to EHLMRS. Among these proteins, some are associated with a primarily neuromuscular disorder (TELT, HSPB8, HXK1, PRPS1) or myopathy (LDHA). Others are linked to dystonia and central nervous system disorders (SPRE), deafness or hearing loss (COX1, SMPX, PRPS1, CAH2, ACY1), encephalopathy (CMC1, VATA, ACY1), various neuro- or neuronopathies (COX1, CATD, HXK1), structural central nervous system anomalies or lesions, partly with visual impairment (COX1, HXK1, PRPS1, ACY1) as well as connective tissue disorders (CO5A1, VATA) (see OMIM number for further, itemized reference). Furthermore, psychomotor retardation and muscle weakness are associated with carbonic anhydrase II deficiency (CAH2) in autosomal recessive osteopetrosis III (OPTB3) [17].

## 3. Discussion

In the present study, we performed the most detailed examination of myopathology associated with SPATA5 deficiency in EHLRMS thus far, described the case of a young girl with EHLMRS and demonstrated an extension of the associated mutational spectrum of the SPATA5 gene [2,6,7,8,9,10,11,12].

Besides the reduced SDH reaction and a low–normal activity of respiratory chain enzymes, hinting towards mitochondriopathy, the extensive work-up, including proteomic analysis, revealed a large selection of dysregulated proteins within the muscle that were localized both in and outside the mitochondrion (Table 1).

The spectrum of diseases known to be associated with many of the significantly dysregulated proteins in our patient includes a variety of neuromuscular, neurodevelopmental and neuropathic disorders, but also isolated features such as deafness or a susceptibility to epilepsy and several diseases for which only core characteristics are known to overlap with the characteristics present in patients with EHLMRS [13,14,15,16,17]. In this respect, the dysregulation of e.g., PURA1, LAMA2 and ECHO could influence the muscular phenotype in EHLMRS as these proteins are known for their involvement in neuromuscular disorders.

Thus, we have delivered additional insights into the molecular etiology of the disease and have provided further evidence for a muscle pathology associated with SPATA5 deficiency, including a pathological histochemical pattern accompanied by dysregulated protein expression. This adds to the previously reported muscle biopsy findings of enlarged and abnormally shaped mitochondria, partly with a subsarcolemmal and intermyofibrillar increase as well as intermyofibrillar mitochondrial aggregates [2]. In light of the fact that, to our knowledge, muscle biopsies of only three SPATA5 patients have been examined, and it is worth noting that all three have shown varying characteristics and that even with normal HE and Gomori trichrome stains, a mitochondrial disorder has to be considered. This adds importance to the examination of proteomic profiles to further delineate a common pathological phenotype. However, given that the proteomically analyzed biopsies also contained cellular populations apart from myofibres, such as fibroblasts, one cannot fully exclude the possibility that the identified protein dysregulations took place within these cells. Moreover, studies on muscle biopsies derived from further SPATA5 cases are needed to define common myopathological hallmarks for this disease entity, which are necessary to align our findings of slightly reduced SDH activity and low–normal respiratory chain enzymes to the phenotypical presentation. Moreover, results of our proteomic profiling suggest extended vulnerability beyond the mitochondria, namely to the extracellular exosome, myosin complex, extracellular matrix (for up-regulated proteins) and the extracellular exosome, cytosol, myelin sheath, endocytic vesicle membrane, extracellular space, endosome membrane, cytoplasm, proteinaceous extracellular matrix and extracellular matrix (for down-regulated proteins).

In conclusion, our results further support the characterization of EHLMRS as a neurodevelopmental disorder with at least mitochondrial affection. How far the proteins up-regulated in muscle compensate for the disease-causing mechanisms, and how far they, together with the down-regulated proteins, are causative for the phenotype remains to be further elucidated.

## 4. Materials and Methods

### 4.1. Exome-Sequencing

Exonic DNA fragments were enriched using a SureSelect Human All Exon kit (Agilent technologies, Santa Clara, CA, USA, 60 Mb V6) for subsequent sequencing on a HiSeq4000 (Illumina, San Diego, CA, USA) to a mean 120-fold coverage as 100 bp paired end reads. Moreover, >98% of the target sequence we covered at least 20-fold. Alignment of reads occurred in the Human Genome Assembly GRCh37 (hg19). Clinical variant prioritization included different filtering steps (in-house-database Munich-Exome-Server, 1000 Genomes, dbSNP, Kaviar, ExAC, gnomAD) as described in Wagner et al. [18] and Stenton et al. [19].

### 4.2. Histology and Enzyme Histochemistry, Immunohistology

Surgically removed sections of muscle tissue were mounted on cork discs with optimal cutting temperature (OCT) compound. Sections were then frozen through inversion of the cork mounted specimen (30 s) in liquid phase isopentane, which was previously cooled in liquid nitrogen (−130 °C) [20]. Cryosections (10 µm) of muscle blocks in transverse orientation were stained according to standard procedures, modified after Dubowitz et al. [20]. Examinations were performed for HE, PAS, modified Gomori trichrome, ATPase 4.3 and 9.4, NADH, COX, SDH and COX-SDH [20,21]. A Zeiss Axioplan epifluorescence microscope equipped with a Zeiss Axio Cam ICc1 was used for light microscopy.

For immunohistology, cryosections (5 µm), prepared as described above, were treated according to the previously described method [22]. Primary antibodies, which were used according to the manufacturers’ specifications, are listed below, and further details can be obtained from the authors upon qualified request (Table 3).

### 4.3. Human Muscle Preparation for Mass Spectrometry

Surgically removed muscle tissue was snap-frozen in liquid nitrogen and stored at −80 °C. In addition to the patient-derived muscle sample (measured in technical triplicates), muscle biopsies of three gender and age-matched individuals were included and defined as non-diseased controls (NDCs). Here, we selected “patients” that had undergone a muscle biopsy due to nonspecific complaints but were found not to have any inflammatory muscle disease, e.g., they suffered from myalgia, but objective muscle weakness and morphological abnormalities on the skeletal muscle biopsy were absent. CK levels were normal and no signs of systemic inflammation, myositis-specific antibodies, (MSA) or myositis-associated antibodies (MAA) were detectable. The total muscle was lysed in 200 µL of 50 mM Tris-HCl (pH 7.8) buffer, 5% SDS, and cOmplete ULTRA protease inhibitor (Roche) using the Bioruptor^®^ (Diagenode) for 10 min (30 s on, 30 s off, 10 cycles) at 4 °C. To ensure complete lysis, an additional sonication step using an ultra-sonic probe (30 s, 1 s/1 s, amplitude 40%) followed by centrifugation at 4 °C and 20,000× *g* for 15 min was completed. Protein concentration of the supernatant was determined by a BCA assay according to the manufacturer’s protocol. Disulfide bonds were reduced by the addition of 10 mM TCEP at 37 °C for 30 min, and free sulfhydryl bonds were alkylated with 15 mM IAA at room temperature (RT) in the dark for 30 min. In each sample, 100 µg protein was used for proteolysis using the S-Trap protocol (Protifi) and using a protein to the trypsin ratio of 20:1. The incubation time for trypsin was changed to 2 h at 37 °C. Proteolysis was stopped using TFA to acidify the sample (pH < 3.0).

All proteolytic digests were checked for complete digestion after desalting by using monolithic column separation (PepSwift monolithic PS-DVB PL-CAP200-PM, Dionex, Germering, Germany) on an inert Ultimate 3000 HPLC (Dionex, Germering, Germany) by direct injection of a 1 μg sample. A binary gradient (solvent A: 0.1% TFA, solvent B: 0.08% TFA, 84% ACN) ranging from 5–12% B in 5 min and then from 12–50% B in 15 min at a flow rate of 2.2 μL/min and at 60°, was applied. UV traces were acquired at 214 nm [23].

### 4.4. DIA-LC-MS/MS Analysis

All samples were analyzed using an UltiMate 3000 RSLC nano UHPLC coupled to a QExactive HF mass spectrometer, with the total amount of peptide applied always being 1 µg. The samples were first transferred to a 75 µm × 2 cm, 100 Å, C18 precolumn with a flow rate of 10 µL/min for 20 min. followed by a separation on the 75 µm × 50 cm, 100 Å, C18 main column with a flow rate of 250 nL/min and a linear gradient consisting of solution A (99.9% water, 0.1% formic acid) and solution B (84% acetonitrile, 15.9% water, 0.1% formic acid), where the pure gradient length was 120 min (3–45% Solution B). The gradient was applied as follows: 3% B for 20 min, 3–35% for 120 min, followed by 3 wash steps each reaching 95% buffer B for 3 min. After the last washing step, the instrument was allowed to equilibrate for 20 min. The acquisition of MS data was performed in DIA (data independent acquisition) mode using an in house build spectral library. Each sample analyzed was mixed with an appropriate amount of iRT Standard IRT (Biognosys, Schlieren, Switzerland). Full MS scans were acquired from 300–1100 *m*/*z* at a resolution of 60,000 (Orbitrap) using the polysiloxane ion at 445.12002 *m*/*z* as lock mass. The automatic gain control (AGC) was set to 3E6 and the maximum injection time was set to 20 milliseconds. Full MS scans were followed by 23 DIA windows, each covering a range of 28 *m*/*z* with 1 *m*/*z* overlap, starting at 400 *m*/*z*, acquired at a resolution of 30,000 (Orbitrap) with an AGC set to 3E6 and nCE of 27 (CID).

### 4.5. Analysis of DIA Data

For the analysis of the samples acquired with nano-LC-MS/MS in DIA mode, the data were introduced to the Spectronaut software (Biognosys, Schlieren, Switzerland) and analyzed with a library-based search. For the library, an in house created spectral library was used. Search and extraction settings were kept as standard (BGS Factory settings). For the proteome background, the human proteome data were selected from UniProt (www.uniprot.org, accessed on 23 July 2018) containing 20,374 entries.

For reliable label-free quantification, only proteins identified with ≥2 unique peptides were considered for further analysis. Following this, the average normalized abundances (obtained by Spectronaut) were calculated for each protein and were used to determine the ratios between patient muscle samples with their respective controls. Lastly, for each protein, log2 transformation of the generated ratios and Student’s *t*-test *p*-values were calculated using MS Excel.

## Figures and Tables

**Table 1:** Dysregulated proteins within the patient’s muscle biopsy showing mitochondrial localization.

UniProt Entry	Gene Name	Patient vs. Control	*p*-Value
HMCS2_HUMAN	*HMGCS2*	6.67	0.000
SPEB_HUMAN	*AGMAT*	2.46	0.000
THIM_HUMAN	*ACAA2*	2.38	0.000
KAD3_HUMAN	*AK3*	1.98	0.001
ATPD_HUMAN	*ATP5F1D*	1.98	0.003
ETFB_HUMAN	*ETFB*	1.95	0.000
ECHA_HUMAN	*HADHA*	1.91	0.000
NDUB6_HUMAN	*NDUFB6*	1.90	0.003
COX5B_HUMAN	*COX5B*	0.54	0.008
CMC1_HUMAN	*SLC25A12*	0.53	0.011
ATP5J_HUMAN	*ATP5J*	0.52	0.009
COX1_HUMAN	*MT-CO1*	0.50	0.026
KAD2_HUMAN	*AK2*	0.50	0.008
CQ10A_HUMAN	*COQ10A*	0.49	0.034
HXK1_HUMAN	*HK1*	0.31	0.000

**Table 2:** Dysregulated proteins and their associated diseases presenting with phenotypical overlap to EHLMRS.

Protein	Localization	Gene Name	*p*-Value ^3^	Associated Disease (#OMIM)
PURA1_HUMAN ^1^	Cytoplasm	*ADSSL1*	0.000	# 617030 MYOPATHY, DISTAL, 5; MPD5
LAMA2_HUMAN ^1^	Extracellular, basement membrane	*LAMA2*	0.013	# 607855 MUSCULAR DYSTROPHY, CONGENITAL MEROSIN-DEFICIENT, 1A; MDC1A
				# 618138 MUSCULAR DYSTROPHY, LIMB-GIRDLE, AUTOSOMAL RECESSIVE 23; LGMDR23
ETFB_HUMAN ^1^	Mitochondrion matrix	*ETFB*	0.000	# 231680 GLUTARIC ACIDEMIA IIB, INCLUDED; GA2B, INCLUDED ETFB DEFICIENCY, INCLUDED
ECHA_HUMAN ^1^	Mitochondrion inner membrane	*HADHA*	0.000	# 609015 MITOCHONDRIAL TRIFUNCTIONAL PROTEIN DEFICIENCY; MTPD
				# 609016 LONG-CHAIN 3-HYDROXYACYL-CoA DEHYDROGENASE DEFICIENCY
				# 231680 MULTIPLE ACYL-CoA DEHYDROGENASE DEFICIENCY; MADD
CMC1_HUMAN ^2^	Mitochondrion inner membrane; multi-pass membrane protein	*SLC25A12*	0.011	# 612949 DEVELOPMENTAL AND EPILEPTIC ENCEPHALOPATHY 39; DEE39
TELT_HUMAN ^2^	Sarcomere	*TCAP*	0.004	# 607487 CARDIOMYOPATHY, FAMILIAL HYPERTROPHIC, 25; CMH25 # 601954 MUSCULAR DYSTROPHY, LIMB-GIRDLE, AUTOSOMAL RECESSIVE 7; LGMDR7
COX1_HUMAN ^2^	Mitochondrion inner membrane; multi-pass membrane protein	*MT-CO1*	0.026	# 535000 LEBER OPTIC ATROPHY
				# 220110 MITOCHONDRIAL COMPLEX IV DEFICIENCY, NUCLEAR TYPE 1; MC4DN1
				# 550500 MYOGLOBINURIA, RECURRENT
				# 500008 DEAFNESS, NONSYNDROMIC SENSORINEURAL, MITOCHONDRIAL
				# 114500 COLORECTAL CANCER; CRC
HSPB8_HUMAN ^2^	Nucleus; cytoplasm	*HSPB8*	0.039	# 158590 NEURONOPATHY, DISTAL HEREDITARY MOTOR, TYPE IIA; HMN2A
				# 608673 CHARCOT-MARIE-TOOTH DISEASE, AXONAL, TYPE 2L; CMT2L
SPRE_HUMAN ^2^	Cytoplasm	*SPR*	0.014	# 612716 DYSTONIA, DOPA-RESPONSIVE, DUE TO SEPIAPTERIN REDUCTASE DEFICIENCY
HEM2_HUMAN ^2^	Cytosol; extracellular exosome; extracellular region; nucleus; ficolin-1-rich granule lumen; secretory granule lumen	*ALAD*	0.031	# 612740 PORPHYRIA, ACUTE HEPATIC
CAH2_HUMAN ^2^	Cell membrane; cytoplasm	*CA2*	0.050	# 259730 OSTEOPETROSIS, AUTOSOMAL RECESSIVE 3; OPTB3
CATD_HUMAN ^2^	Lysosome; extracellular space; melanosome	*CTSD*	0.003	# 610127 CEROID LIPOFUSCINOSIS, NEURONAL, 10; CLN10
LDHA_HUMAN ^2^	Cytoplasm	*LDHA*	0.009	# 612933 GLYCOGEN STORAGE DISEASE XI; GSD11
HXK1_HUMAN ^2^	Cytosol; mitochondrion outer membrane; peripheral membrane protein	*HK1*	0.000	# 235700 HEMOLYTIC ANEMIA, NONSPHEROCYTIC, DUE TO HEXOKINASE DEFICIENCY
				# 605285 NEUROPATHY, HEREDITARY MOTOR AND SENSORY, RUSSE TYPE; HMSNR
				# 617460 RETINITIS PIGMENTOSA 79; RP79
				# 618547 NEURODEVELOPMENTAL DISORDER WITH VISUAL DEFECTS AND BRAIN ANOMALIES; NEDVIBA
SMPX_HUMAN ^2^	Nucleus; costamere; M band—muscle tendon junction	*SMPX*	0.036	# 300066 DEAFNESS, X-LINKED 4; DFNX4
VATA_HUMAN ^2^	Cytoplasm	*ATP6V1A*	0.006	# 617403 CUTIS LAXA, AUTOSOMAL RECESSIVE, TYPE IID; ARCL2D
				# 618012 DEVELOPMENTAL AND EPILEPTIC ENCEPHALOPATHY 93; DEE93
PRPS1_HUMAN ^2^	Cytosol; cytoplasm—ribose phosphate diphosphokinase complex	*PRPS1*	0.040	# 300661 PHOSPHORIBOSYLPYROPHOSPHATE SYNTHETASE SUPERACTIVITY
				# 311070 CHARCOT-MARIE-TOOTH DISEASE, X-LINKED RECESSIVE, 5; CMTX5
				# 301835 ARTS SYNDROME; ARTS
				# 304500 DEAFNESS, X-LINKED 1; DFNX1
CO5A1_HUMAN ^2^	Extracellular matrix	*COL5A1*	0.007	# 130000 EHLERS-DANLOS SYNDROME, CLASSIC TYPE, 1; EDSCL1
ACY1_HUMAN ^2^	Cytoplasm	*ACY1*	0.002	# 609924 AMINOACYLASE 1 DEFICIENCY; ACY1D

^1^ Proteins showing significant up-regulation. ^2^ Proteins showing significant down-regulation. ^3^ Expression patient vs. control.

**Table 3:** Primary antibodies used in immunohistology.

Primary Antibody, Clone	Company	Number	Dilution
Caveolin 3, 26/Caveolin3	BD Transduction Laboratories	610420	1:500
Dystrophin 1, Dy4/6d3	Novocastra/Leica	NCL-DYS1	1:2
Dystrophin 2, Dy8/6c5	Novocastra/Leica	NCL-DYS2	1:10
Dystrophin 3, Dy10/12B2	Novocastra/Leica	NCL-DYS3	1:10
α-Dystroglycan, VIA4-1	Merck/Upstate	#05-298	1:15
β-Dystroglycan, 43DAG1/8D5	Novocastra/Leica	NCL-b-DG	1:10
α-Sarcoglycan, Ad1/20A6	Novocastra/Leica	NCL-a-SARC	1:50
β-Sarcoglycan, βSarc/5B1	Novocastra/Leica	NCL-b-SARC	1:50
γ-Sarcoglycan, 35DAG/21B5	Novocastra/Leica	NCL-g-SARC	1:50
δ-Sarcoglycan, 35DAG/21B5	Novocastra/Leica	NCL-d-SARC	1:10
Laminin α2 C-term., 5H2	Merck/Chemicon	MAB1922	1:500
Emerin, 4G5	Novocastra/Leica	NCL-EMERIN	1:20
Dysferlin, Hamlet 1	Novocastra/Leica	NCL-HAMLET	1:5
Collagen 6, VI-26	Merck/Chemicon	MAB3303	1:100
Collagen 6, 3C4	Merck/Chemicon	MAB1944	1:200
Collagen 4, polyclonal	Cedarline	CL50411AP	1:100
Laminin α5, 4C7	Merck/Chemicon	MAB1924	1:500
DRP 2 (Utrophin), DRP3/20C5	Novocastra/Leica	NCL-DRP2	1:5
Neonatal Myosin, WB-MHCn	Novocastra/Leica	NCL-MHCn	1:5

## Data Availability

Data of the proteomic profiling can be accessed through the ProteomeXchange project accession # PXD026182.

## Supplementary Materials

The following are available online at https://www.mdpi.com/article/10.3390/ijms22157835/s1.
