# Supplementary material for: Muscular and Molecular Pathology Associated with SPATA5 Deficiency in a Child with EHLMRS
Source: Int J Mol Sci. 2021 Jul 22;22(15):7835. doi: 10.3390/ijms22157835 (PMC8345956; doi:10.3390/ijms22157835)
Supplement: Supplementary file 1 [file ijms-22-07835-s001.zip › ijms-1289616-supplementary.pdf]

Table S1. Dysregulated proteins within patient muscle biopsy showing significant up-regulation (a) or down-regulation (b).

a)

UniProt Entry	Gene name	Patient vs Control	p-value
IQGA1_HUMAN	IQGAP1	16,76	0,001
XIRP2_HUMAN	XIRP2	8,38	0,015
MY18A_HUMAN	MYO18A	7,38	0,005
HMCS2_HUMAN	HMGCS2	6,67	0,000
PRVA_HUMAN	PVALB	3,54	0,001
STIM1_HUMAN	STIM1	3,46	0,022
MYH11_HUMAN	MYH11	3,23	0,000
CKAP4_HUMAN	CKAP4	2,82	0,016
ADIPO_HUMAN	ADIPOQ	2,66	0,000
EIF3E_HUMAN	EIF3E	2,47	0,031
SPEB_HUMAN	AGMAT	2,46	0,000
GSTM1_HUMAN	GSTM1	2,43	0,000
THIM_HUMAN	ACAA2	2,38	0,000
CAVN4_HUMAN	CAVIN4	2,36	0,015
PSMD8_HUMAN	PSMD8	2,36	0,003
CAD13_HUMAN	CDH13	2,34	0,000
VPS4A_HUMAN	VPS4A	2,27	0,003
HSPB3_HUMAN	HSPB3	2,26	0,000
NAR3_HUMAN	ART3	2,24	0,000
PURA1_HUMAN	ADSSL1	2,17	0,000
LAMA2_HUMAN	LAMA2	2,05	0,013
PPR3A_HUMAN	PPP1R3A	2,05	0,016
COFA1_HUMAN	COL15A1	2,04	0,008
HSPB2_HUMAN	HSPB2	2,00	0,021
KAD3_HUMAN	AK3	1,98	0,001
ATPD_HUMAN	ATP5F1D	1,98	0,003
ETFB_HUMAN	ETFB	1,95	0,000
DNJA2_HUMAN	DNAJA2	1,94	0,004
RD23B_HUMAN	RAD23B	1,93	0,001
ECHA_HUMAN	HADHA	1,91	0,000
NDUB6_HUMAN	NDUFB6	1,90	0,003

b)

UniProt Entry	Gene name	Patient vs Control	p-value
COX5B_HUMAN	COX5B	0,54	0,008
ACYP2_HUMAN	ACYP2	0,54	0,000
CMC1_HUMAN	SLC25A12	0,53	0,011
TELT_HUMAN	TCAP	0,53	0,004
UBB_HUMAN;UBC_HUMAN;RS27A_HUMAN;RL40_HUMAN	UBB;UBC;RPS27A;UBA52	0,52	0,034
		0,52	0,046
CO6A6_HUMAN	COL6A6	0,52	0,009

ATP5J_HUMAN	ATP5J	0,52	0,021
AT1B1_HUMAN	ATP1B1	0,51	0,003
MGST3_HUMAN	MGST3	0,51	0,016
GSTO1_HUMAN	GSTO1	0,50	0,003
POSTN_HUMAN	POSTN	0,50	0,026
COX1_HUMAN	MT-CO1	0,50	0,008
KAD2_HUMAN	AK2	0,49	0,034
CQ10A_HUMAN	COQ10A	0,46	0,006
AMPD1_HUMAN	AMPD1	0,45	0,028
AL1A1_HUMAN	ALDH1A1	0,45	0,014
FHOD1_HUMAN	FHOD1	0,44	0,033
TCPA_HUMAN	TCP1	0,42	0,039
HSPB8_HUMAN	HSPB8	0,41	0,007
CUL5_HUMAN	CUL5	0,40	0,014
SPRE_HUMAN	SPR	0,39	0,009
CALR_HUMAN	CALR	0,39	0,005
SBP1_HUMAN	SELENBP1	0,39	0,031
HEM2_HUMAN	ALAD	0,38	0,042
SMTL1_HUMAN	SMTNL1	0,38	0,018
HYEP_HUMAN	EPHX1	0,38	0,001
TWF2_HUMAN	TWF2	0,38	0,021
PROB1_HUMAN	PROB1	0,38	0,000
DUS3_HUMAN	DUSP3	0,37	0,050
ALDOC_HUMAN	ALDOC	0,36	0,050
CAH2_HUMAN	CA2	0,36	0,003
CATD_HUMAN	CTSD	0,36	0,001
PLCD4_HUMAN	PLCD4	0,34	0,009
LDHA_HUMAN	LDHA	0,31	0,000
HXK1_HUMAN	HK1	0,29	0,036
SMPX_HUMAN	SMPX	0,28	0,000
RU17_HUMAN	SNRNP70	0,26	0,015
SYPL1_HUMAN	SYPL1	0,25	0,029
IGHA2_HUMAN	IGHA2	0,25	0,025
GPX3_HUMAN	GPX3	0,25	0,005
IDI2_HUMAN	IDI2	0,24	0,039
ACTY_HUMAN	ACTR1B	0,24	0,000
GSTM3_HUMAN	GSTM3	0,24	0,006
VATA_HUMAN	ATP6V1A	0,19	0,017
FRIH_HUMAN	FTH1	0,18	0,040
PRPS1_HUMAN	PRPS1	0,17	0,000
NDKB_HUMAN	NME2	0,14	0,007
CO5A1_HUMAN	COL5A1	0,12	0,045
H15_HUMAN	HIST1H1B	0,11	0,002
ACY1_HUMAN	ACY1		
